# A contemporary survey of bumble bee diversity across the state of California

**DOI:** 10.1002/ece3.8505

**Published:** 2022-03-18

**Authors:** Kaleigh Fisher, Kristal M. Watrous, Neal M. Williams, Leif L. Richardson, Sarah Hollis Woodard

**Affiliations:** ^1^ Department of Entomology University of California, Riverside Riverside California USA; ^2^ Department of Entomology and Nematology University of California, Davis Davis California USA; ^3^ The Xerces Society for Invertebrate Conservation Portland Oregon USA

**Keywords:** biodiversity, bumble bees, California, conservation

## Abstract

Bumble bees (genus *Bombus*) are important pollinators with more than 260 species found worldwide, many of which are in decline. Twenty‐five species occur in California with the highest species abundance and diversity found in coastal, northern, and montane regions. No recent studies have examined California bumble bee diversity across large spatial scales nor explored contemporary community composition patterns across the state. To fill these gaps, we collected 1740 bumble bee individuals, representing 17 species from 17 sites (~100 bees per site) in California, using an assemblage monitoring framework. This framework is intended to provide an accurate estimate of relative abundance of more common species without negatively impacting populations through overcollection. Our sites were distributed across six ecoregions, with an emphasis on those that historically hosted high bumble bee diversity. We compared bumble bee composition among these sites to provide a snapshot of California bumble bee biodiversity in a single year. Overall, the assemblage monitoring framework that we employed successfully captured estimated relative abundance of species for most sites, but not all. This shortcoming suggests that bumble bee biodiversity monitoring in California might require multiple monitoring approaches, including greater depth of sampling in some regions, given the variable patterns in bumble bee abundance and richness throughout the state. Our study sheds light on the current status of bumble bee diversity in California, identifies some areas where greater sampling effort and conservation action should be focused in the future, and performs the first assessment of an assembly monitoring framework for bumble bee communities in the state.

## Graphical abstract

Bumble bees are important pollinators worldwide, with 25 species occurring in California. We collected 1,740 bees at 17 sites across California, the first regional study in California in nearly 40 years. Our study sheds light on the current status of bumble bee diversity in California and identifies areas where greater sampling and conservation action should be targeted in the future.

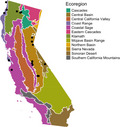

## 1 INTRODUCTION

Standardized species monitoring frameworks are critical for documenting population trends and managing their conservation (Lindenmayer & Likens, 2010; Nichols & Williams, 2006). Intensive ecological monitoring is predicted to be extremely costly and time intensive for bees (Portman & Tepedino, 2021; but see Breeze et al., 2021), which are highly diverse (>4,000 species in North America; Michener, 2007), difficult to count and identify in situ, and which overwhelmingly lack baseline data of populations at biologically relevant scales (Woodard et al., 2020). Thus, calls have been made to identify ways to make bee monitoring more effective, streamlined, and also cognizant of potential overcollection (Portman et al., 2020; Tepedino et al., 2015). The latter is particularly important to minimize overcollection of already‐threatened species. Bumble bees (genus *Bombus*, family Apidae) are an ecologically important group for which monitoring is desperately needed and which are experiencing substantial declines worldwide (Goulson et al., 2008; Williams & Osborne, 2009). An estimated approximately one‐third of species in North America are considered to be in decline by the International Union for the Conservation of Nature (IUCN) (Arbetman et al., 2017; Hatfield et al., 2014). Testing whether standardized, conservation‐minded monitoring frameworks can be applied broadly across regions is particularly important for developing robust monitoring frameworks for bumble bees and other threatened bee groups. Ideally, these frameworks can successfully document assemblage diversity patterns and ultimately be employed to inform their conservation and management.

The state of California encompasses the majority of the Mediterranean‐climate California Floristic Province, one of the world's top 25 biodiversity hotspots (Howell, 1957; Mittermeier et al., 1999). Given its high levels of biodiversity and species endemism, the state is a critical target of global conservation efforts (Myers et al., 2000). California is among the states most impacted by global changes such as rapid urbanization and development, agricultural intensification, and climate change (CDFW, 2015), which are threatening biodiversity throughout the state. Among its diverse and threatened taxa, California is home to 25 bumble bee species (Hymenoptera: Apidae, *Bombus* Latreille), which equates to approximately 50% of all North American species (Williams et al., 2014) and ~10% of those worldwide (Williams, 1998). Bumble bees provide important pollination services to both crop and noncrop plants, which is important for ecosystem function (Velthuis & van Doorn, 2006). The genus *Bombus* is comprised of approximately 260 species globally. It includes the social subgenera, which have a reproductive caste that includes males and females and a nonreproductive female worker caste, as well as the socially parasitic subgenus *Psithyrus* Lepeletier, which only produces reproductive males and females. Six California bumble bee species are currently considered of conservation concern within the state by the California Department of Fish and Wildlife (CDFW): *B*. *caliginosus* (Frison), *B*. *crotchii* Cresson, *B*. *franklini* (Frison), *B*. *morrisoni* Cresson, *B*. *occidentalis* Greene, *and B*. *suckleyi* Greene (CNDDB, 2020). These species are also considered threatened across their entire ranges by IUCN, where three are assessed as vulnerable (*B*. *caliginosus*, *B*. *morrisoni*, *B*. *occidentalis*), one as endangered (*B*. *crotchii*), and two as critically endangered (*B*. *franklini*, *B*. *suckleyi*) (Hatfield et al., 2014).

Efforts to characterize the current status of bumble bee populations in California are necessary for establishing baseline information about relative abundance, continuing to develop more refined species range maps, and ultimately conserving this pollinator group in the context of the state's rapidly changing landscapes and climate. However, developing and testing standardized bumble bee‐monitoring frameworks in California is especially challenging for two reasons. First, in contrast to much of the United States, where the majority of the bumble bee nesting season is contained within the summer months, the nesting season of bumble bee species in California can occur all year long, depending on the species (Williams et al., 2014). Thus, it can be difficult to predict the optimal timeframes for sampling a given area. Moreover, bumble bees are found in vastly distinct habitats across the state (Williams & Osborne, 2009), with highly variable densities depending on the habitat (Thorp et al., 1983). For example, they tend to be most abundant and speciose in northern, coastal, and montane areas, and much less so in the Central Valley and Southern California, especially at lower elevation sites in the Mojave and Sonoran Deserts (Thorp et al., 1983). As a result, monitoring frameworks that collect a consistent number of individuals across sites might not accurately describe patterns of diversity and abundance in California, as they do in other parts of the United States (Strange & Tripodi, 2019).

Contemporary studies of bumble bees have been performed in targeted areas of California, including parts of the Sierra Nevada Mountains (Hatfield & LeBuhn, 2007; Loffland et al., 2017) and urban areas along the coast (McFrederick & LeBuhn, 2006; Schochet et al., 2016). Additional studies have examined entire bee communities (including bumble bees) in specific areas in the state, such as the decades‐long bee‐monitoring effort at Pinnacles National Park (Meiners et al., 2019). However, the last statewide analysis of bumble bee biodiversity in California was performed in the early 1980s by Thorp et al. (1983), and there have been no other statewide studies since that time. Habitat loss, disease, and climate change, among other stressors, which are strongly influencing bumble bee species abundances and distributions worldwide (Cameron, Lozier, et al., 2011; Goulson et al., 2015; Kerr et al., 2015; Sirois‐Delisle & Kerr, 2018), also appear to be impacting bumble bee persistence in California. Several threatened bumble bee species began to decline precipitously in the late 1990s and early 2000s (Cameron, Lozier, et al., 2011; Graves et al., 2020; Hatfield et al., 2014; Koch & Strange, 2009; Thorp, 2005), leading to the six designated species of conservation concern in the state today. However, more subtle changes in species' ranges, community compositions, and phenology cannot currently be assessed, given that there have been no contemporary, large‐scale efforts to assess bumble bee species assemblage composition in the state since these declines began.

**FIGURE 1:**
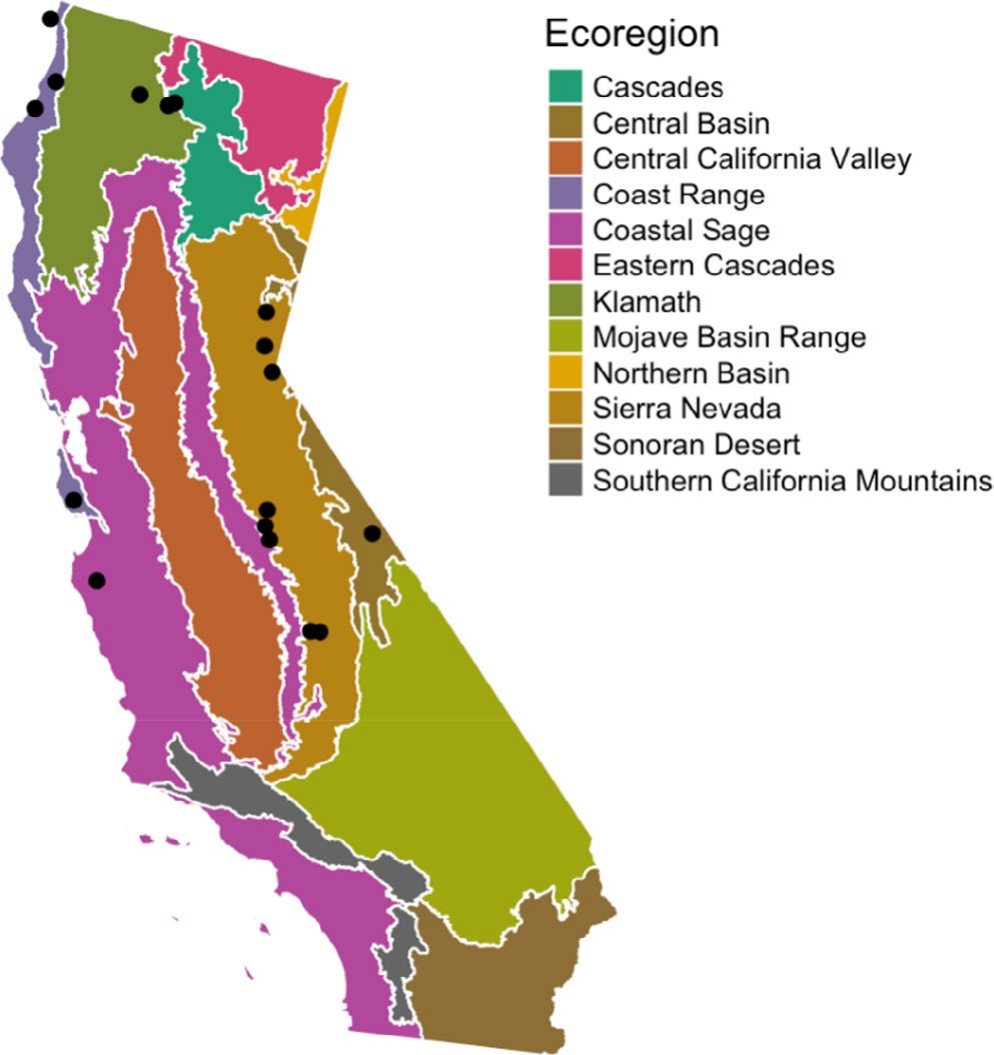
Map of the 17 sites sampled across 6 of the 13 Level III Ecoregions in California

To address the lack of current information about the status of California bumble bees, we performed sampling of bumble bees across a wide geographic range in the state during the 2019 nesting season. In our study, we followed an assemblage monitoring framework modeled after Strange and Tripodi (2019), who assessed bumble bee communities across most of the United States (albeit not in California). Use of this protocol allowed us to maximize our ability to assess general trends in bumble bee community composition across our sites, while minimizing negative impacts on bumble bee populations through overcollection. Based on an analysis of 31 sites across the United States, this approach is highly informative for inferring bumble bee assemblage composition at individual sites at particular time points (Strange & Tripodi, 2019), as well as for identifying sampling dates to collect certain species. This information is critical to develop effective, larger scale monitoring efforts. In our study, we collected bees from a set of 17 sites distributed across the state of California, with an emphasis on the Coast Range, Klamath, and Sierra Nevada ecoregions (Figure 1). These areas are part of the California Floristic Province biodiversity hotspot and have historically harbored the most diverse bumble bee communities in the state (Thorp et al., 1983).

Many monitoring protocols tend to prioritize rare and threatened species in order to assess their conservation statuses and inform efforts to conserve them. However, monitoring is also needed for more common species so that they can also be targeted for conservation efforts if they begin to decline, in order to prevent substantial declines in the future and avoid extinction debt (Kuussaari et al., 2009). Our 1‐year study provides a general update on statewide trends in bumble bee community composition, focusing on more common species, and also tests whether a standardized, assemblage monitoring framework can be applied consistently across biologically and geographically diverse states like California.

## 2 MATERIALS AND METHODS

### 2.1 Site selection and sampling

Collection sites were opportunistically selected based on presence of bumble bee‐visited plants, accessibility (by vehicle and/or by foot), permitted access, and distribution across the state and ecoregions, with an emphasis on the coastal and montane regions that are most speciose (Thorp et al., 1983). We aimed to sample at sites with peak worker abundance, which is typically before production of male and gyne offspring. Bees were collected from areas no larger than 10 km^2^ and these sampling sites were >12 km apart. We assigned all of our sampling sites to their EPA Level III Ecoregion classifications (Griffith et al., 2016; Strange & Tripodi, 2019) in accordance with practices of the California Department of Fish and Wildlife (CDFW) and U.S. Fish and Wildlife Service (USFWS). We collected all specimens during spring and summer 2019 (May–August). We aimed to collect approximately 100 individuals at each site over one to three consecutive collection days and between the hours of 1,000–1,800, following methods of Strange and Tripodi (2019). All bees were collected on days with no rain or high winds. We collected bees primarily from flowers; when possible, we recorded associated plant species data to inform future monitoring efforts but did not systematically document floral resource availability at sites. Bees were collected into 70% ethanol and then stored at −80°C to preserve tissue for future analyses.

### 2.2 Bumble bee identification

Following collection, all bees were removed from ethanol (with a single leg retained in ethanol as a DNA voucher), rinsed with ethanol, dried, then pinned. Bees were identified to species following diagnostic characteristics in Thorp et al. (1983), Stephen (1957), and Williams et al. (2014), and were retained by the Woodard Lab at UC Riverside. We refer to some species listed in Thorp et al. (1983) with updated taxonomic nomenclature following Williams et al. (2014): *B*. *californicus* now as *B*. *fervidus*, *B*. *sonorus* as *B*. *pensylvanicus*, *B*. *fernaldae* as *B*. *flavidus*, *B*. *edwardsii* as *B*. *melanopygus*, *B*. *balteatus* as *B*. *kirbiellus* (Williams et al., 2019), *B*. *bifarius* species complex as *B*. *bifarius* (Ghisbain et al., 2020). We used a combination of morphological characters and geography to distinguish between two very similar species, *B*. *vosnesenskii* and *B*. *caliginosus*. *Bombus caliginosus* is almost entirely restricted to the coast ranges near the Pacific Ocean (Koch et al., 2012; Thorp et al., 1983), and does not occur east of the Central Valley (Williams et al., 2014). In contrast, *B*. *vosnesenskii* is more broadly distributed throughout the state. In sites where the pair could occur in sympatry, we distinguished them based on morphological characteristics from Williams et al. (2014). Specifically, in *B*. *vosnesenskii*, the malar space is not longer than wide; T4 (T = tergum) is completely yellow; S3–4 (S = sternum) have only black hairs; and there are many large pits on the lower central area of the clypeus. In *B*. *caliginosus*, the malar space is longer than wide; the leading edge of T4 has many black hairs medially; S3–4 have yellow hairs; and there are only small or only a few large pits on the lower central area of the clypeus. In all cases, identification based on morphological characteristics was consistent with our conception of the two species distributions (e.g., no bees resembling *B*. *caliginosus* were detected outside of Coast Range sites). Bee species identities, floral associations, and associated data are available through Dryad. All specimen data have been deposited in the CDFW's California Natural Diversity Database.

### 2.3 Statistical methods

All statistical analyses were performed using R version 4.0.2 (R Core Team, 2020). We estimated study‐wide relative abundance for each species by dividing the total number of each species across all sampling sites by the total number of bees collected across all sampling sites. To estimate species richness and Shannon (H′) diversity for each site and ecoregion, we generated sample size‐based rarefaction and extrapolation curves with 95% confidence intervals using the iNEXT package (v. 2.0.2; Hsieh et al., 2016).

We used nonmetric multidimensional scaling analyses (NMDS) with a Bray–Curtis dissimilarity index to visualize similarity in bee assemblages between sites (Minchin, 1987; Warton et al., 2012). This was followed by an analysis of similarity (ANOSIM) in the Vegan package (v. 2.5–6; Oksanen et al., 2019) to test whether there were differences in species composition between ecoregions. The ANOSIM compared the mean distance between sites within an ecoregion to the mean distance among sites between ecoregions based on species clustering patterns (Clarke, 1993). We performed the NMDS and ANOSIM on (1) abundance of every species at each site, and (2) presence–absence data at each site, in order to explore whether similarities or differences in bee assemblages between sites were due to the abundance of shared species or species identity (Williams, 2011).

To contextualize our species richness results with species historically found in the sampled ecoregions, we also obtained historical bumble bee specimen data from 35 public and private collections, assembled by L.L. Richardson (Richardson, 2020; Williams et al., 2014; Table 2). This database includes a substantial fraction of the specimen material examined by Thorp et al. (1983), among other museum collections, and is the best available digital repository of historical records in California (See Appendix A for complete list of collections). We did not directly compare the current survey data to historical records quantitatively because of major differences in sampling approach and coverage. Instead, this comparison provides an index of individual species' persistence in California ecoregions over time.

## 3 RESULTS

We collected a total of 1,740 individual bumble bees across 17 collection sites (mean number of bees per site = 102 ± SE 2; range = 84–110) in 6 different ecoregions (Figure 1). No bees were collected in Southern California; we attempted to collect at 4 sites in Southern California but could not locate more than 10 bees per site at the time of our visits. Our resulting dataset includes 17 different *Bombus* species, representing 68% of bumble bee species known to inhabit California historically and 34% of the approximately 50 US bumble bee species. Twenty‐eight percent of all bees collected were males and the remainder were females of the worker caste, or females of parasitic species. This indicates that although we aimed to collect bees at peak worker abundance, we collected at several sites toward the end of the nesting season.

### 3.1 Common and rare species in the dataset

The most commonly collected species was *B*. *vosnesenskii*, which represented more than half (~57%) of all collected bees. We collected this species in all six ecoregions we sampled, and at all of our sites except for one in the Sierra Nevada ecoregion (Site NS2). With respect to relative abundance, this species was followed only distantly by *B*. *melanopygus*, which represented 8% of all bees collected and was collected at nine sites across four ecoregions (Table 1).

**TABLE 1:** Summary table for species

Species	Sites	Ecoregions	Total relative abundance %
*B. vosnesenskii* Radoszkowski	16	6	57
*B*. *melanopygus* Nylander	9	4	8
*B*. *bifarius* Cresson	6	3	7
*B*. *flavifrons* Cresson	5	3	7
*B*. *rufocinctus* Cresson	11	5	4
*B*. *mixtus* Cresson	4	2	4
*B*. *caliginosus* (Frison)	5	2	2
*B*. *huntii* Greene	1	1	2
*B*. *vandykei* (Frison)	5	2	1
*B*. *flavidus* ^a^ Eversmann	4	4	1
*B*. *fervidus* (Fabricius)	3	3	1
*B*. *insularis* ^a^ (Smith)	3	2	1
*B*. *kirbiellus* Curtis	1	1	1
*B*. *sylvicola* Kirby	1	1	3
*B*. *centralis* Cresson	1	1	0.3
*B*. *griseocollis* (De Geer)	1	1	0.2
*B*. *appositus* Cresson	1	1	0.1

^a^ Indicates subgenus *Psithyrus*.

The rarest species in our dataset were *B*. *appositus* (*n* = 2 specimens collected), *B*. *centralis* (*n* = 5), and *B*. *griseocollis* (*n* = 3). Each of these species was collected at only a single site, and each from a unique ecoregion (Sierra Nevada, Central Basin, and Coast Range, respectively). Four species were collected exclusively from one site in the Central Basin: the high‐elevation species *B*. *kirbiellus* (*n* = 12), *B*. *centralis* (*n* = 5), *B*. *huntii* (*n* = 28), and *B*. *sylvicola* (*n* = 48). We detected two species in the socially parasitic subgenus *Psithyrus*: *B*. *insularis* was collected at three of our sites in the Sierra Nevada and Cascades ecoregions (*n* = 21 bees collected across all sites) and *B*. *flavidus* was collected at four of our sites in the Sierra Nevada, Cascades, Klamath, and Coast Range ecoregions (*n* = 23 bees collected across all sites).

All 17 of the species we collected have historically been reported in the same ecoregions where we collected them (Table 2). However, many of the species we collected in only a subset of the ecoregions where they have been found historically. For example, *B*. *caliginosus*, the only imperiled species we observed, was collected at five sites (*n* = 36 bees). Although this species was historically present in four of the ecoregions we sampled, we only collected it in two ecoregions, Coastal Sage and Coast Range (Table 2).

**TABLE 2:** Contemporary (2019) and historical species richness by Ecoregion

Ecoregion	# Sites sampled (2019)	Total species richness (2019)	Historical species richness	Species
Cascades	1	6	20	*appositus*, ***bifarius***, *caliginosus* ^a^, *centralis*, *fervidus* **, *flavidus***, *flavifrons*, *franklini* ^a^, *griseocollis*, ***insularis*,** *melanopygus*, *mixtus*, *morrisoni*, *nevadensis*, *occidentalis* ^a^, ***rufocinctus*,** *suckleyi* ^a^, *sylvicola*, ***vandykei*, *vosnesenskii***
Central Basin	1	7	18	*appositus*, *bifarius*, ***centralis***, *crotchii* ^a^, ***fervidus***, *flavifrons*, *griseocollis*, ***huntii***, *insularis*, ***kirbiellus***, *melanopygus*, *mixtus*, *morrisoni* ^a^, *nevadensis*, *pensylvanicus*, *rufocinctus* **, *sylvicola*, *vosnesenskii***
Coastal Sage	1	4	21	*appositus*, *bifarius*, ***caliginosus*** ^a^, *centralis*, *crotchii*, ***fervidus***, *flavidus*, *flavifrons*, *griseocollis*, *huntii*, *insularis*, ***melanopygus***, *mixtus*, *nevadensis*, *occidentalis* ^a^, *pensylvanicus*, *rufocinctus*, *sitkensis*, *sylvicola*, *vandykei*, ***vosnesenskii***
Coast Range	4	9	19	*bifarius*, ***caliginosus*** ^a^, *centralis*, *crotchii* ^a^, ***fervidus*, *flavidus***, *flavifrons*, ***griseocollis*,** *huntii*, *insularis*, ***melanopygus***, ***mixtus***, *occidentalis* ^a^, *pensylvanicus*, ***rufocinctus***, *sitkensis*, *vandykei*, ***vosnesenskii***
Klamath	2	6	21	***bifarius*** , *caliginosus*, *crotchii* ^a^, *fervidus* **, *flavidus***, ***flavifrons***, *franklini* ^a^, *insularis*, *griseocollis*, *huntii*, ***melanopygus*,** *mixtus*, *morrisoni* ^a^, *occidentalis* ^a^, ***rufocinctus***, *sitkensis*, *suckleyi* ^a^, *sylvicola*, *vandykei*, ***vosnesenskii***
Sierra Nevada	8	10	22	*appositus*, ***bifarius***, *centralis*, *crotchii* ^a^, *fervidus*, ***flavidus*, *flavifrons***, *griseocollis*, *huntii*, ***insularis***, *kirbiellus*, ***melanopygus*, *mixtus***, *morrisoni* ^a^, *nevadensis*, *occidentalis* ^a^, *pensylvanicus*, ***rufocinctus*,** *sitkensis*, *sylvicola*, ***vandykei*, *vosnesenskii***

Note

Number of bees collected per site averaged 102 ± 1.5 SE (Range: 84–110).

Bold indicates species that were found in 2019 and historically.

Unbolded indicates species only found historically.

^a^ Considered imperiled by CDFW.

### 3.2 Bumble bee species richness

**FIGURE 2:**
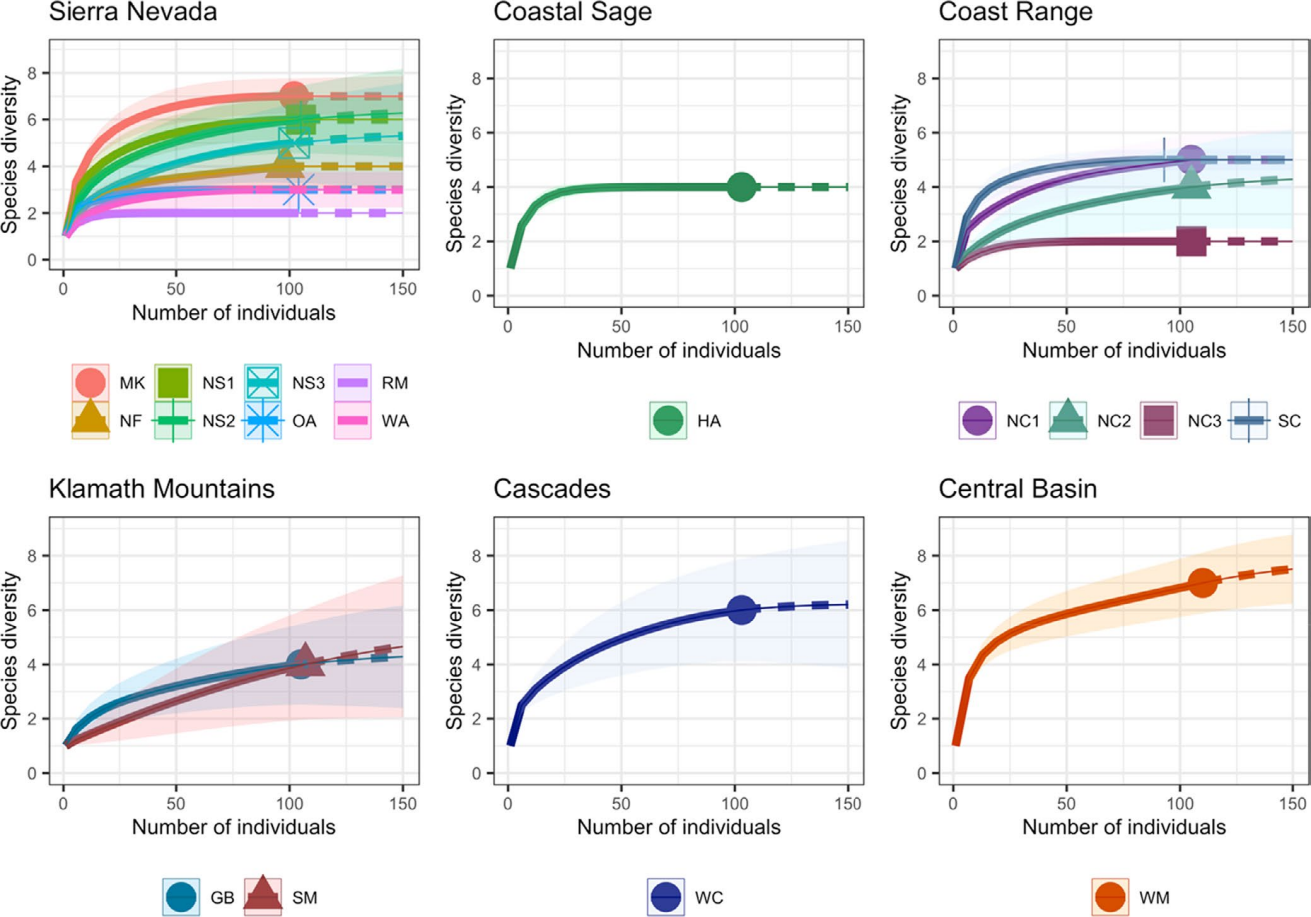
Rarefaction curves generated from estimated species richness for each site according to Level III Ecoregion. Extrapolation estimates are based on sampling 150 specimens

Estimated species richness was comparable to observed species richness for nearly all of the sites (15 of 17). For 15 sites, our estimates of species richness reached asymptotes and extrapolation did not predict that we would observe more species with greater sampling effort (Figure 2). The two exceptions were one site in the Central Basin (eight species estimated; seven species observed) and one site in the Klamath Mountains (six species estimated; four observed). We detected higher species diversity in ecoregions where we sampled at more sites, such as the Sierra Nevada and Coast Range (Table 2). The Central Basin and the Sierra Nevada had sites with the highest alpha diversity (WM; MK; Richness = 7) followed by the site in the Cascades (WC; Richness = 6). The Sierra Nevada also had a site with the lowest alpha diversity (RM; Richness = 2), in addition to a site in the Coast Range (NC3; Richness = 2).

### 3.3 Community composition

**FIGURE 3:**
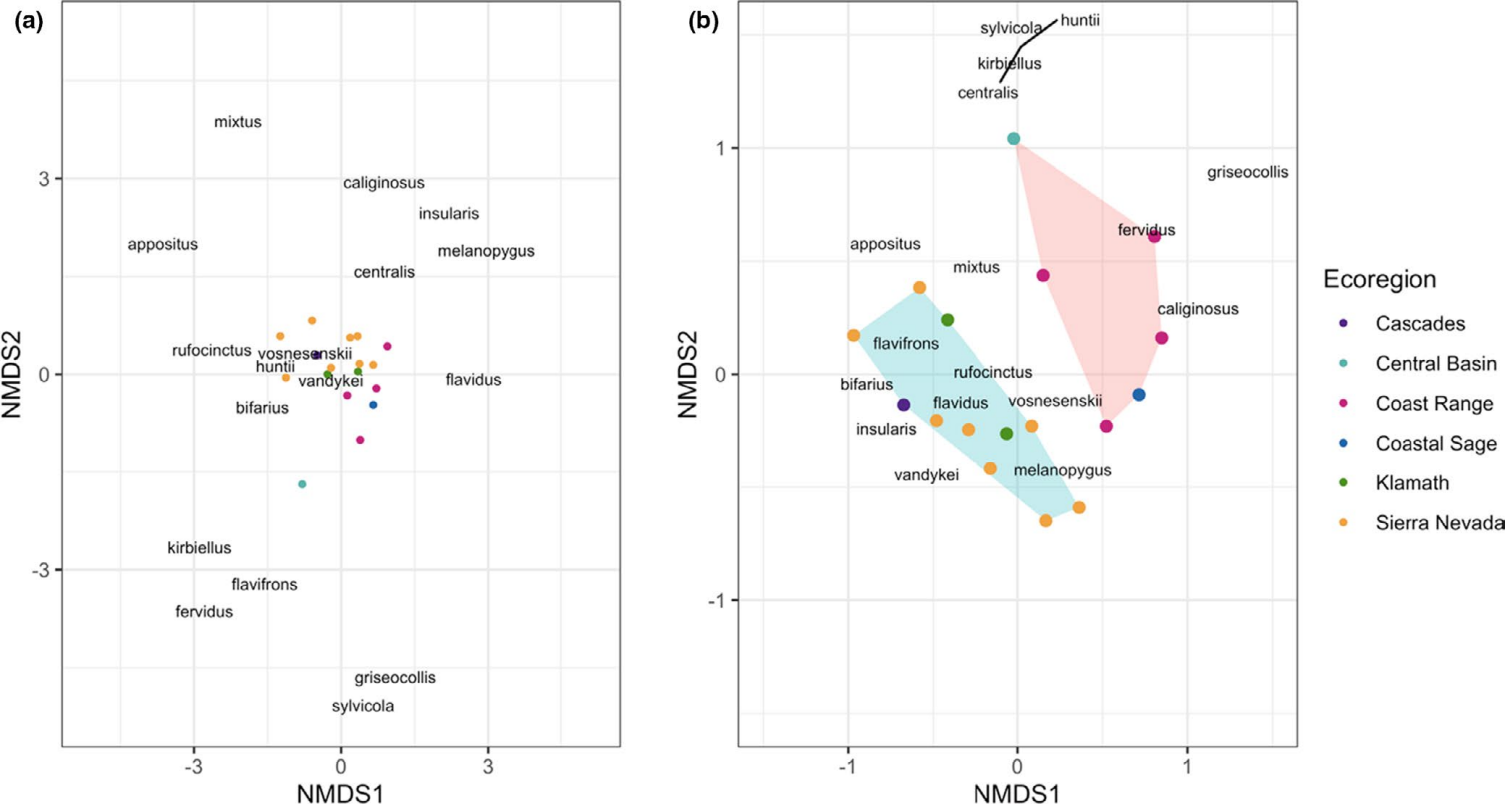
Nonmetric multidimensional scaling of study sites using (a) species abundance and identity and (b) presence–absence of species. Ordinations were based on Bray–Curtis dissimilarity. Circles represent sites, which are clustered relative to species shared between sites (indicated with species name). Lines in B indicate shared location for *B. centralis*, *B. huntii*, *B. kirbiellus*, and *B. sylvicola*; species names are offset for better visualization

There were no differences in community composition among ecoregions when we considered both species abundance and identity (Figure 3a; *R* ^2^ = .983; stress = 0.13; ANOSIM Ecoregion: *R* = .0611, *p* = .34). However, differences between ecoregions were detected when we considered only species identity (Figure 3b; *R* ^2^ = .985; stress = 0.121; ANOSIM Ecoregion: *R* = .3909, *p* = .0078). We found two distinct ecoregion groupings: Group One included the higher elevation, mountainous ecoregions Klamath, the Sierra Nevada, and the Cascades, whereas Group Two included the lower elevation ecoregions Coastal Sage, Coast Range, and the Central Basin (ANOSIM Group: *R* = .5747, *p* < .001). There were no significant differences in composition based on species identity between the ecoregions within Group One (ANOSIM Ecoregion Group One: *R* = −.04377, *p* = .5669) or Group Two (ANOSIM Ecoregion Group Two: *R* = .3704, *p* = .3333).

### 3.4 Caste distribution

**FIGURE 4:**
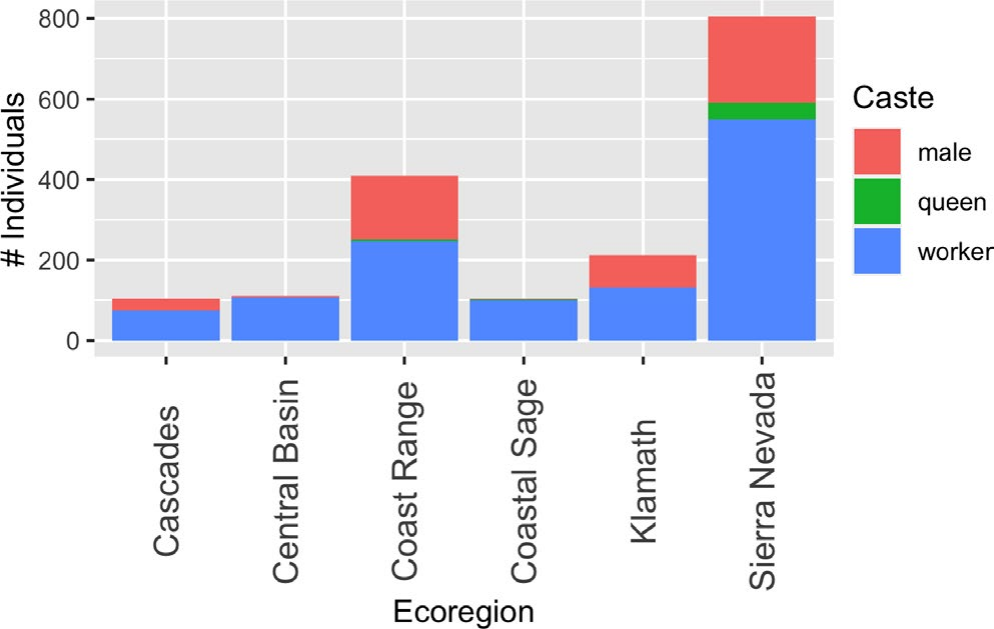
Distribution of workers, queens, and males among individuals collected in each ecoregion. The presence of queens indicates earlier stage colonies, while the presence of males indicates later stage colonies

We collected workers and males at all sites, and queens at eight sites. Workers made up more than 50% of individuals collected at the majority of sites (*N* = 12), and more than half of individuals collected at each ecoregion (Figure 4). We collected mostly males at the five sites where a majority of workers were not collected, which indicates that at these sites, bumble bees were approaching the end of their colony cycle at our collection time points.

## 4 DISCUSSION

Our study provides the first broad overview of California bumble bee species diversity in nearly 40 years (Thorp et al., 1983). During this period, California has undergone considerable landscape‐level changes due to agricultural intensification, urbanization, and climate change (CDOC, 2021), all of which affect bumble bee habitat, making our study particularly timely. We sampled a fairly consistent number of individuals (84–110) at each of our 17 sites, but we had an uneven representation of sites among ecoregions (range of 1–8 sites per ecoregion). In the Sierra Nevada and Coast Range, where we collected at more sites, we detected higher ecoregion‐level diversity, even though some sites in these ecoregions had the lowest alpha diversity overall. Our results provide a modern overview of bumble bee richness throughout the state and can be used as a contemporary baseline to guide future monitoring efforts.

Our rarefaction analyses show that data collected with the assemblage monitoring framework we employed was generally representative of site species richness. However, the standardized framework we used should be modified in the following ways: (1) include a wider range of sampling months to accommodate the phenology of California's bumble bees, and sample sites repeatedly to capture species turnover; (2) collect more individuals in sites that have historically harbored greater diversity to capture all species predicted to be present based on species accumulation estimates; and (3) conduct species‐specific targeted sampling of Southern California bumble bees to assess species trends where the collection of 100 specimens is not feasible, as well as targeted sampling of rarer species in their historical ranges.

Bumble bee species differ phenologically, and the peak abundance of workers occurs at different times throughout the foraging season depending on the species (Goulson, 2003; Williams et al., 2014). In some ecoregions we sampled a preponderance of males, whose presence indicates the approach of or end of a flight season for a species (Goulson, 2003). Although we aimed to collect at sites that were mid‐season for the most bumble bee species, we may have missed the peak abundance of some of the species we did not collect, but were expecting to find. Our caste distribution data indicate that future sampling can begin at similar dates to when we sampled in the Central Basin, Coastal Sage, and Cascades, where we collected a majority of workers. However, given that we collected a high proportion of males in the Coastal Range, Klamath, and Sierra Nevada, future sampling should begin earlier than the dates when we sampled.

Our species accumulation analysis suggests that the sampling framework generally captured the bumble bee assemblage present at our sites during the time of sampling. This is consistent with what was found by Strange and Tripodi (2019) in their assessment of bumble bee communities across the United States, which focused on the Eastern United States. However, two exceptions to this pattern in our study include the site in the Central Basin, and one site in the Klamath Mountains, where estimated richness exceeded observed. Given that we collected more than 100 bees at both of these sites, our collection numbers alone do not explain our failure to reach asymptotes in this analysis. These ecoregions have historically harbored high numbers of bumble bee species, in addition to hosting species that are rare in other ecoregions in California. For example, *B*. *kirbiellus* was historically present at high elevations in the Central Basin and Sierra Nevada, but we only observed it in the Central Basin. *Bombus suckleyi* has historically only been observed in the Cascades and in the Klamath Mountains (Thorp et al., 1983; Table 2). Our finding that the assemblage monitoring framework used by Strange and Tripodi (2019) can be employed for most places we sampled in California, but not all, suggests that a single framework with uniform effort at a single time point might not be applicable across the state of California, given its high levels of habitat and species heterogeneity. Rather, it suggests that more intensive, or more targeted, sampling may be required for ecoregions that have historically harbored rarer species.

For the species that were found historically in a region, that we did not collect, it is unclear whether they were not collected because (i) they are rare and require greater sampling to detect, (ii) they require sampling at a different time point in the season, or (iii) they are no longer present in an area. Thus, our comparison between historical and contemporaneous (our) data must be interpreted with caution. Nonetheless, of the eight species that we did not collect in our study, five are of conservation concern according CDFW and the IUCN (*B*. *occidentalis*, *B*. *suckleyi*, *B*. *franklini*, *B*. *crotchii*, and *B*. *morrisoni*). Thus, their increasing rarity may partially explain why we did not collect them in our study. *Bombus occidentalis* was historically widespread across the state (Thorp et al., 1983), but is now restricted to high meadows (CDFW, 2019; Graves et al., 2020). This species appears to still be sporadically present in the Sierra Nevada (Cole et al., 2020; Hatfield & LeBuhn, 2007), and the Northern Coast Range (Graves et al., 2020), although we did not sample it in either of these ecoregions, which is consistent with another recent study in the Sierra Nevada (Loffland et al., 2017). The documented decline of *B*. *occidentalis* began in the mid‐1990s in the most western parts of its range, including in California (Cameron, Jepsen, et al., 2011; Graves et al., 2020). The decline of *B*. *occidentalis* is also a driving factor in the decline of *B*. *suckleyi*, a socially parasitic species that depends on *B*. *occidentalis* as its host (Lhomme & Hines, 2019; Thorp et al., 1983). Species at higher trophic levels, including social parasites, are more vulnerable to decline than those at lower trophic levels (de la Mora et al., 2020; Klein et al., 2006). *Bombus franklini* was last observed in California in 1998 and in Oregon in 2006, and is considered Critically Endangered by the IUCN (CDFW, 2019). This species has the smallest known range of any bumble bee species and was historically restricted to southern Oregon and Northern California (Plowright & Stephen, 1980; Thorp, 2005; Williams et al., 2014).

We may not have collected *B*. *crotchii* and *B*. *morrisoni*, as well as the remaining three species that are not currently considered to be threatened (*B*. *nevadensis*, *B*. *sitkensis*, and *B*. *pensylvanicus*), because of the timing and/or location of our sampling, rather than their extreme rarity. All of these species are present in recent California observations reported to online community science databases (e.g., iNaturalist, Bumble Bee Watch). Moreover, although we sampled in ecoregions where all of these species were historically present (Cole et al., 2020; Thorp et al., 1983; Table 2), we had a limited number of sites in the ecoregions where *B*. *nevadensis* (Cole et al., 2020; Thorp et al., 1983) and *B*. *morrisoni* have previously been collected (CDFW, 2019; Graves et al., 2020; Thorp et al., 1983; Table 2). Similarly, the current range of *B*. *crotchii* and *B*. *pensylvanicus*, which were previously abundant throughout California, appears to now be restricted to xeric and coastal sites in Southern California (Schweitzer et al., 2012; Thorp et al., 1983; Richardson & Woodard, unpublished data) and the Central Valley, where we did not sample.

Sampling in additional regions of the state should also be included in future monitoring efforts to provide a more detailed picture of California bumble bee species distributions. These include, for example, the Southern California mountains and the Central California foothills. The Central Valley and the southeastern part of the state, where bumble bees tend to be less abundant (Thorp et al., 1983), should also be targeted for extensive sampling to detect rarer species like *B*. *crotchii* and *B*. *pensylvanicus*. Sampling may need to be especially targeted in Southern California, as we were not able to collect ~100 individuals from any site in this region. Results from more comprehensive sampling can then be rigorously compared to historical bumble bee distributions, and statistical analysis can be performed to shed light on the status and trends of bumble bee populations in the state.

*Bombus caliginosus* was the only threatened species we observed during our sampling. Although this species is considered threatened in the state by CDFW, our data suggest that it is potentially less rare than commonly assumed in the coastal areas where it occurs, given that this species comprised up to 16.5% of the community in some sites where we sampled. *Bombus caliginosus* is easily mistaken for *B*. *vosnesenskii* (Stephen, 1957; Thomson, 2016), making differentiating between the two species in coastal regions challenging. We propose that, specifically in the mountainous coastal areas of California where their distributions overlap, the occurrence of *B*. *caliginosus* might be underestimated because it is mistakenly identified as the vastly more common *B*. *vosnesenskii*, especially in nondestructive sampling studies.

The widespread distribution of four dominant species likely explains why community composition did not differ among ecoregions, when we performed this analysis using species abundance metrics. *Bombus vosnesenskii* is highly abundant throughout California and was the dominant species across all of our sites, consistent with previous studies of statewide patterns by Thorp et al. (1983) and in the Sierra Nevada by Loffland et al. (2017). A similar pattern, albeit with a unique species, was observed by Strange and Tripodi (2019), who sampled largely in the Eastern United States and found that a single highly common species, *B*. *impatiens*, was dominant across their sites. In our study, *B*. *vosnesenskii* represented more than half of the specimens collected and was present at all of our sites except one in the Sierra Nevada. Similarly, *B*. *melanopygus*, *B*. *bifarius*, and *B*. *flavifrons*, which had the three next highest relative abundances after *B*. *vosnesenskii*, were found in more than half of the ecoregions where we sampled. The rarer species that turned over among sites and regions did not influence abundance‐weighted metrics of community similarity. When we only considered species presence or absence, however, we revealed significant differences in composition between ecoregions. Thus, rarer species we collected likely contribute most to the beta diversity among ecoregions, although greater depth of sampling is needed to confirm this. Species turnover, or beta diversity, is the proportion of species composition that changes between sites. Similar patterns have been found in other analyses of spatial beta diversity in bees (Winfree et al., 2018) as well as in other taxa (Cardinale et al., 2011; Isbell et al., 2011). When considered in terms of presence–absence, higher elevation sites appeared to have greater species similarity compared to the lower elevation sites. The differences between these two groups does not necessarily indicate greater similarity within each group. This is exemplified by the Central Basin having four unique species, even though there were no significant differences between the Central Basin and coastal ecoregions. Moreover, we only sampled at one site in the Central Basin, and species turnover may be detected with greater sampling. Sampling at more sites in the Central Basin could thus potentially yield higher ecoregion‐level richness and thus more pronounced differences in species composition compared to the other ecoregions.

Our study extends Strange and Tripodi's (2019) sampling to California and largely validates the efficacy of their approach, which can be employed to capture information about the bumble bee community at a site while minimizing harmful impacts on local populations from overcollecting. However, our study has several limitations that need to be considered when drawing conclusions from our results. First, as discussed above, we sampled an uneven number of sites in each ecoregion, which prevented us from explicitly estimating species turnover between sites. Second, our sampling framework, which is designed to minimize overcollection, may not have been sensitive to rarer taxa, which are often missed with standardized efforts that do are not specifically designed to inventory rare species. As a result, we may have collected rare species in only a subset of the ecoregions where they were actually present. Finally, limiting collection to a single season in 1 year precluded phenological turnover and phenologically driven variation in abundance or years. Each of these limitations likely prevented us from capturing full ecoregional diversity and important spatial variation. Our collections provide a critical snapshot of the relative abundance and diversity among sites across multiple ecoregions in California and is a necessary first step to develop future monitoring efforts. Specifically, species occurrences can be used to inform the dates and locations selected for more comprehensive sampling.

Key barriers to successfully implementing species‐specific conservation actions include the lack of large‐scale monitoring studies to identify general patterns, as well as knowledge gaps in life history and drivers of species decline (Graves et al., 2020; USFWS, 2019). Overcoming these barriers and protecting important species, like bumble bees, is necessary to prevent cascading negative impacts on agricultural and natural ecosystems (CDFA, 2018; Cole et al., 2020; Kremen et al., 2002; Macior, 1977; Thorp, 2014). Our study highlights the need to assess standardized monitoring frameworks for species assemblages with high habitat and species heterogeneity. Specifically, our study shows that greater monitoring of the diverse bumble bees of California is needed in order to better understand the drivers of biodiversity and decline in this genus, and to more effectively manage bumble bee conservation in the state.

## CONFLICT OF INTEREST

None.

## AUTHOR CONTRIBUTIONS

**Kaleigh Fisher:** Data curation (supporting); formal analysis (lead); investigation (lead); visualization (lead); writing – original draft (lead); writing – review and editing (lead). **Kristal M. Watrous:** Conceptualization (supporting); data curation (lead); investigation (supporting); methodology (supporting); project administration (supporting); writing – review and editing (equal). **Neal Williams:** Conceptualization (supporting); funding acquisition (supporting); project administration (supporting); writing – review and editing (equal). **Leif Richardson:** Formal analysis (supporting); investigation (supporting); resources (supporting); validation (supporting); writing – review and editing (equal). **S. Hollis Woodard:** Conceptualization (lead); data curation (supporting); formal analysis (supporting); funding acquisition (lead); investigation (lead); methodology (lead); project administration (lead); resources (lead); writing – original draft (equal); writing – review and editing (equal).

## Data Availability

Data and associated scripts for data analysis can be accessed through Dryad: https://doi.org/10.6086/D1FT3H.

## APPENDIX A

Collections contributing bumble bee specimen records used for the historical comparison. The American Museum of Natural History dataset includes digitized records from a variety of secondary sources.

**Table:**

Institution	Number of specimens
Essig Museum of Entomology, UC Berkeley	10,697
USDA‐ARS	6,534
LA County Museum	2,281
Institute for Bird Populations	2,242
Bohart Museum of Entomology, UC Davis	1,782
U.C. Riverside Entomology Museum	862
Snow Entomological Museum, University of Kansas	700
Kenneth S. Norris Center for Natural History, UC Santa Cruz	616
American Museum of Natural History	591
Smithsonian	585
California State Collection of Arthropods	504
Illinois Natural History Survey (INHS)	357
California Academy of Sciences	242
Yale University Peabody Museum	211
Texas A&M University	146
N. Williams Research Collection	137
University of Central Florida Collection of Arthropods	88
Field Museum of Natural History	54
University of Colorado Museum of Natural History, Boulder, CO	49
Occidental College	38
C.A. Triplehorn Insect Collection, Ohio State University	26
York University	23
North Carolina State University Insect Museum	12
El Colegio de la Frontera Sur	11
Harvard	8
Cornell University Insect Collection	7
Cleveland Museum of Natural History	4
UC San Diego	4
Frost Entomological Museum Penn State	3
Research Collection of Paul H. Williams	2
R. E. Irwin Research Collection	2
Wisconsin Insect Research Collection	2
Diane Thomson Research Collection	2
Royal Ontario Museum	1
Humboldt State University Insect Laboratory	1
Total specimens	28,280
